# Synergistic effect of *APOE4* genotype and *ELOVL2* methylation on cognitive slowing and verbal memory decline in non‐demented adults during aging

**DOI:** 10.1002/alz70856_107609

**Published:** 2026-01-09

**Authors:** Natalya V Ponomareva, Tatiana V Andreeva, Irina L Kuznetsova, Maria Protasova, Daria Malina, Svetlana Kunizheva, Daria Kupryanova, Victoria Petrova, Ekaterina P Kolesnikova, Andrey Mitrofanov, Vitaly Fokin, Sergey N. Illarioshkin, Evgeny I Rogaev

**Affiliations:** ^1^ Research Center of Neurology, Moscow, Other, Russian Federation; ^2^ Vavilov Institute of General Genetics of Russian Academy of Sciences, Moscow, Other, Russian Federation; ^3^ Center for Genetics and Life Science, Sirius University of Science and Technology, Sochi, Russian Federation; ^4^ Vavilov Institute of General Genetics of Russia Academy of Sciences, Moscow, other, Russian Federation; ^5^ Center for Genetics and Life Science, Sirius University of Science and Technology, Sochi, Russian Federation; ^6^ Research Center of Neurology, Moscow, Russian Federation; ^7^ Research Center of Mental Health, Moscow, Other, Russian Federation; ^8^ Department of Psychiatry, Umass Chan Medical School, Shrewsbury, MA, USA; ^9^ Center for Genetics and Life Science, Sirius University of Science and Technology, Sochi, Other, Russian Federation

## Abstract

**Background:**

Aging plays a crucial role in exacerbating the negative impact of the *APOE4* genotype on brain function, heightening the risk of Alzheimer disease (AD). Methylation of the *ELOVL2* promoter is a robust epigenetic biomarkers of age. *ELOVL2* is involved in the production of *long*‐chain *polyunsaturated fatty acids*, which regulate synaptic plasticity and white matter integrity. P3 component of event‐related potentials (ERPs) elicited by the odd‐ball paradigm is a reliable marker of cognitive processing. This study investigated the associations between *APOE* genotype, *ELOVL2* methylation and characteristics of the ERP P3 component and verbal memory in non‐demented adults during aging

**Methods:**

We examined 75 non‐demented volunteers, age range 20‐84 years, 44 APOE4‐ and 31 *APOE4+*. Methylation of *ELOVL2* promoter was measured in the blood. Auditory ERPs were recorded using the odd‐ball paradigm, and memory was assessed using the Luria verbal memory test. Informed written consent was obtained from all participants. All subjects underwent a neurological examination.

**Results:**

P3 latency was positively correlated with *ELOVL*2 methylation, and the correlation remained significant when age was statistically controlled, implying that epigenetic mechanisms might contribute to the prolongation of ERP latency. The correlation between *ELOVL*2 methylation and P3 latency was higher in *APOE*4 carriers than in noncarriers. The decrease of verbal memory during aging was more pronounced in *APOE4* carriers than in non‐carriers. The partial correlation analysis controlling for age revealed a significant correlation between *ELOVL*2 methylation and verbal memory in *APOE4+* carriers only. Moreover, ERP P3 latency was inversely correlated with verbal memory scores, and this correlation was stronger in *APOE4+* carriers.

**Conclusion:**

Impaired lipid metabolism linked to synaptic dysfunction and deterioration of white matter integrity, driven by age‐dependent *ELOVL2* methylation, may accelerate the impact of the *APOE4* genotype on neurophysiological slowing during aging. These changes may contribute to verbal memory decline in *APOE4+* carriers and increase the risk of AD.

**Funding**: This study was supported by Russian Science Foundation (Project 19‐75‐30039 to TA genotyping; Project 22‐15‐00448 to NP, VF, EK ERP analysis and MP for genotyping) and by Ministry of Science and Higher Education of the Russian Federation (Agreement 075‐10‐2021‐093 project GEN‐RND‐2017).

